# Reusability of autoclaved 3D printed polypropylene compared to a glass filled polypropylene composite

**DOI:** 10.1186/s41205-021-00111-x

**Published:** 2021-08-09

**Authors:** Kristin M. Fischer, Andrew P. Howell

**Affiliations:** 1 Biology Department, Hampden-Sydney College, PO Box 33, VA 23943 Hampden Sydney, USA; 2 Biochemistry & Molecular Biology Department, Hampden-Sydney College, 23943 Hampden Sydney, VA USA

**Keywords:** Sustainability, Fused deposition modelling, Fused filament fabrication, Additive Manufacturing, Material extrusion, Mechanical properties, Medical device

## Abstract

Health care waste can be a costly expenditure for facilities as specific disposal methods must be used to prevent the spread of pathogens. If more multi-use medical devices were available, it could potentially relieve some of this burden; however, sterilization between uses is important in preventing disease transmission. 3D printing has the ability to easily create custom medical devices at a low cost, but the majority of filaments utilized cannot survive steam sterilization. Polypropylene (PP) can withstand autoclave temperatures, but is difficult to print as it warps and shrinks during printing; however, a composite PP filament reduces these effects. Commercially available PP and glass filled PP (GFPP) filaments were successfully 3D printed into 30 × 30 × 30 mm cubes with no shrinking or warping and were autoclaved. The 134 °C autoclave temperature was too high as several cubes melted after two to three rounds, but both PP and GFPP cubes displayed minimal changes in mass and volume after one, four, seven, and ten rounds of autoclaving at 121 °C. GFPP cubes autoclaved zero, four, seven, and ten times had significantly smaller average compressive stress values compared to all PP groups, but the GFPP cubes autoclaved once were only less than PP cubes autoclaved zero, seven and ten times. GFPP cubes autoclaved zero, one, four, and seven times also deformed less indicating that the embedded glass fibers provided additional strength. While a single method was found that successfully printed PP and GFPP cubes that were able to survive up to ten rounds of autoclaving, future work should include further investigation into the mechanical properties and increasing the number of autoclave rounds.

## Background

The World Health Organization (WHO) defines health care waste as the “by-product of health care that includes sharps, non-sharp blood contaminated items, blood, body parts and tissues, chemicals, pharmaceuticals, and radioactive materials” [1]. It is common for health care facilities in developed nations to have their infectious waste transported by a third party either before or after being treated using autoclaving, microwaving, or incineration prior to disposal [2, 3]. The disposal of health care waste has impacts both financially and environmentally as safe handling precautions must be taken to prevent the spread of pathogens [2, 4, 5]. In addition, the high cost associated with this type of disposal may incentivize illegal medical waste treatment or dumping and pocketing the resulting funds [2].

One way to increase sustainability in the health care field is to focus on creating more multi-use medical devices rather than single use devices to decrease the impact of health care waste [4, 6]. Money et al. recommends using human factors engineering methods to create a high quality and well-designed medical device that takes into account the specific needs of the user [7]. This design approach should also take into account the ability of the materials to be sterilized and the sterilization methods available to the user to increase reusability. The majority of medical devices are made of materials that can be sterilized using steam to destroy microorganisms to prevent disease [8–12]. Some advantages of steam sterilization include that it is nontoxic to the patients, staff, and the environment, the cycle is easy to control and monitor, and it is an efficient and speedy process [8, 10]. Additionally, many health care facilities have autoclaves in-house that can be easily used for steam sterilization of medical devices due to its relatively inexpensive cost [8, 11]. The two most common cycles are 121 °C for 30 min or 132 °C for 4 min [8, 10]. However, not all materials used for medical device manufacturing can withstand the autoclave process.

3D printing also known as additive manufacturing, material extrusion, fused deposition modelling (FDM), and fused filament fabrication (FFF) is when a thermoplastic filament is melted, extruded through a circular nozzle, deposited using a 3-axis system onto a print bed, and the 3D object fabricated layer-by-layer from a computerized model [13–25]. 3D printing is an attractive option for medical device fabrication due to its ability to fabricate potentially inexpensive custom pieces over a relatively short time period; however, the two most common 3D printing filaments, poly(lactic acid) (PLA) and acrylonitrile butadiene styrene (ABS), are not suitable because neither can withstand the necessary temperatures for autoclave sterilization [15, 22, 23, 26–29]. Polypropylene (PP) has been gaining traction as a 3D printing material due its low cost, chemical resistance, moisture stability, high impact strength, and use in medical devices [13, 18–20, 22, 28, 30]. PP has a melting temperature above the necessary autoclave temperatures; however, 3D printed PP has been shown to warp and shrink during the layer-by-layer fabrication process due to its semicrystalline nature [13, 16, 18–20, 22, 28, 31, 32].

PP composites using both natural and synthetic fillers like cellulose, hemp fiber, harakeke fiber, bamboo fiber, carbon fiber, PLA, ABS, and glass have been created to decrease the shrinkage and warpage that occurs during the 3D printing process and increase the mechanical properties. The PP composites had varying effects on alleviating the shrinkage and warpage, but the majority showed some degree of improvement with the addition of the filler to the PP filament as it provided more stability and rigidity [13, 14, 16–19, 21, 28, 29, 31, 33, 34]. The goal of this paper was to compare the ability of commercially available PP filament to commercially available glass filled PP (GFPP) filament to 3D print rectangular objects and survive several rounds of autoclaving with no melting or loss in mechanical strength. The hypothesis was that the GFPP fiber would better 3D print rectangular objects and have increased mechanical strength, but would be more likely to melt during the autoclaving process compared to PP 3D printed ones.

## Methods

### 3D printing

A FlashForge® Creator Pro 3D printer with FlashPrint© 3D software was initially used with Verbatim_TM_ PP and Kehuashina® GFPP 1.75 mm filaments. These filaments were extruded through a brass nozzle or extruder with a 0.4 mm diameter to fabricate 20 × 20 × 10 mm rectangular prisms with an open triangular or hexagonal interior and a covered exterior. The rectangular prisms were designed using Tinkercad® modelling software. As PP adheres better to itself, several overlapping strips of 3 in PP packing tape were applied to the stock print bed until it was fully covered and contained no bubbles or excess creases [13, 14]. Several attempts at successfully printing PP and GFPP were needed and Table 1 lists the 3D printing parameters used.

**Table 1:** 3D printing parameters for PP and GFPP using FlashPrint© software

Print Number	Filament Type	Print Speed (mm/s)	Travel Speed (mm/s)	Print Head Temperature (°C)	Print Bed Temperature (°C)	Shell Layer (mm)	Layer Height (mm)	Infill
1	PP	40	60	230	105	1.2	0.16	8 % Open triangle
2	PP	50	70	240	60	1.2	0.16	8 % Open triangle
3	GFPP	60	80	245	60	1.6	0.10	8 % Open triangle
4	GFPP	50	80	245	105	Top & Bottom: 2.4 Perimeter: 2.0	Base: 0.27 Perimeter: 0.22	10 % Open triangle
5	PP & GFPP	50	80	245	105	Top & Bottom: 2.4 Perimeter: 2.0	0.22	10 % Open hexagon

**Fig. 1:**
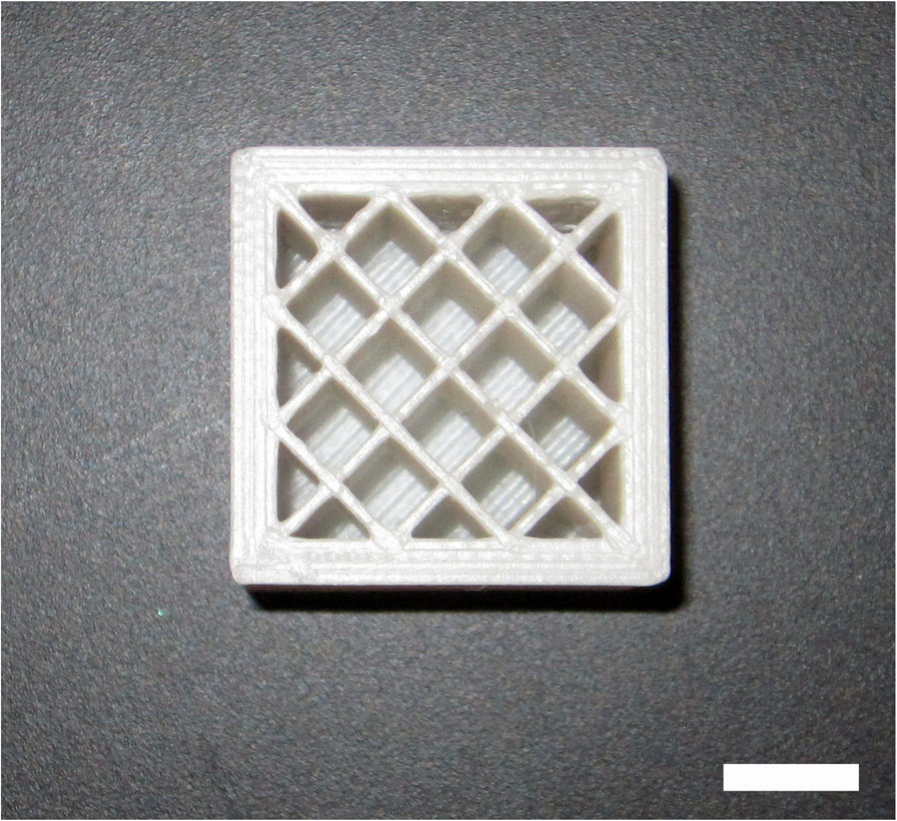
A partially printed cube using the parameters listed in Table 2 displaying the open rectangular grid interior and the thicker exterior walls. Scale bar represents 10 mm

Several changes to the 3D printing process were made to continue to increase the success of printing PP and GFPP rectangular objects. These changes included using Simplify3D® software because there were more printing parameters available, substituting a larger glass print bed made by Greenlee3D for the stock print one, changing the print bed size in the Simplify3D® software accordingly, covering the new larger glass print bed with a single strip of 7 in PP packing tape that contained no creases or bubbles, and using a PrimaCreator hardened steel 0.6 mm nozzle. Due to the fact that the Verbatim_TM_ PP 1.75 mm filament was currently unavailable, FlashForge® PP 1.75 mm filament was used for the rest of the study. The Simplify3D® software came with several presets and Table 2 displays whether the preset PP software settings were used or the new values and options enabled. All PP and GFPP 30 × 30 × 30 mm cubes with an open rectangular grid interior and covered exterior were modelled in Tinkercad®. All cubes were 3D printed using the method described above with the chamber fully enclosed from here on out. Figure 1 displays a partially printed cube that shows the open rectangular grid and thicker exterior walls.

**Table 2:** 3D printing parameters for PP and GFPP filament using the Simplify3D® software

Filament Type	PP & GFPP
Speeds	• Printing speed: Software preset • Travel speed: Software preset • Outline under speed: 60 % • Solid infill under speed: 80 %
Print Head Temperature (°C)	240
Print Bed Temperature (°C)	105
Shell Layer (mm)	Top & Bottom: 3 Perimeter: 1.8
Layer Height (mm)	0.2
Infill	25 % Open rectangular grid
Fan Speed	Layer 1: 10 % All other layers: Software preset
Extruder	Extrusion width: auto Extrusion multiplier: Software preset
Other Enabled Parameters	• Raft to protect the base layer and increase print bed adhesion • Nozzle retraction • Nozzle coast at end • Wipe nozzle

### Pre-autoclave characterization and autoclaving procedures

PP and GFPP 30 × 30 × 30 mm cubes as described above with the raft removed were randomly grouped into either the no autoclave control, one round of autoclaving, four rounds of autoclaving, seven rounds of autoclaving, or ten rounds of autoclaving with five cubes of PP and GFPP assigned to each group. Each cube was marked for the number of rounds of autoclaving and a value from one to five on one of the side faces. For example, PP cube 1,3 was the third cube in the PP group that was autoclaved once. PP and GFPP cubes that were to undergo autoclaving were weighed to obtain their initial mass (*m*_*i*_). Values for the same polymer in the same group were reported as the average ± standard deviation. For initial volume (*v*_*i*_), water displacement was used first; however, the cubes floated possibly due to the open rectangular grid interior rendering this method unusable (Fig. 1). Instead, the initial volume for each individual cube was obtained by measuring the labelled side cube face across the center to determine x and y measurements. The z measurement was taken across the center of the top cube face and the three values multiplied together for each cube to be autoclaved.

PP (n = 20) and GFPP (n = 20) cubes were placed in sterilization pouches with five cubes inside each pouch and autoclaved (Tuttnauer® 3870EA) using the preset program of 134 °C for 7 min with a 45 min drying time. The sterilization pouches were removed and allowed to cool to 22–23 °C before the next round of autoclaving commenced if needed. A second set of 40 cubes was also placed into sterilization pouches with five cubes inside each pouch and autoclaved (Tuttnauer® 3870EA) at 121 °C with a 30 min sterilization time followed by a 30 min drying time. Once again, the sterilization pouches were removed, cooled to 22–23 °C, and autoclaved again if needed. The remaining 20 cubes served as the no autoclave control cubes and were categorized as PP0 or GFPP0 respectively.

### Post-autoclave characterization

**Fig. 2:**
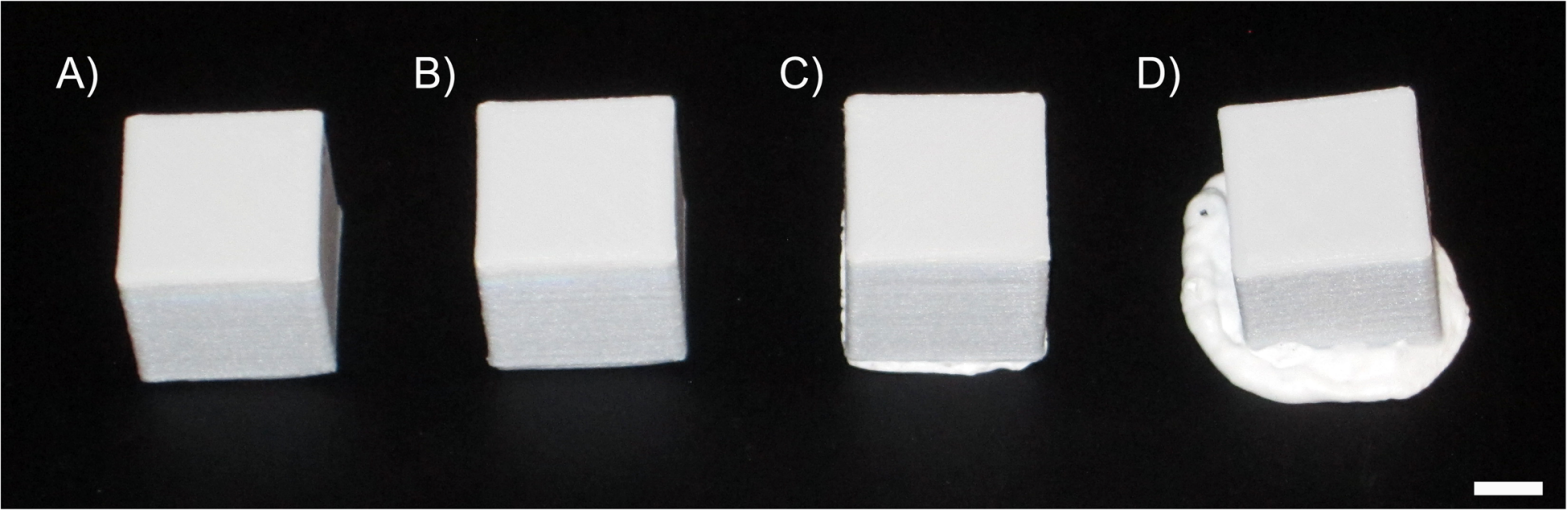
Representative images of 30 x 30 x 30 mm cubes for **A**) category 0 displaying no volumetric loss, **B**) category 1 displaying <1% volumetric loss, **C**) category 2 with a volumetric loss ranging from 1-4%, and **D**) category 3 with >4% volumetric loss. Scale bar represents 10 mm

After all cubes were cooled to room temperature, each was weighed for a final mass (*m*_*f*_) and the percent mass loss using Equation (Eq.) 1 was calculated for each cube individually. These values were reported as the average ± standard deviation for each group. The final volume (*v*_*f*_) for each individual cube was determined as described above and the percent volume loss for each cube was calculated using Eq. 2. These values were reported as the average ± standard deviation for each group. In addition, a 0 to 3 grading scale was established that aimed to categorize the cubes based on the percent volume loss that occurred after autoclaving (Table 3) and Fig. 2 displays representative images for each of the four categories.

**Table 3:** Grading scale for melting post-autoclaving using the calculated percent volumetric loss

Category	Description
0	No volumetric loss
1	< 1 % volumetric loss
2	1-4 % volumetric loss
3	> 4 % volumetric loss

1 $$\%\ mass\ loss=\left(\frac{{m}_{i}-{m}_{f}}{{m}_{i}}\right)*\left(100\right)$$

2 $$\%\ volume\ loss= \left(\frac{{v}_{i}-{v}_{f}}{{v}_{i}}\right)*\left(100\right)$$

**Fig. 3:**
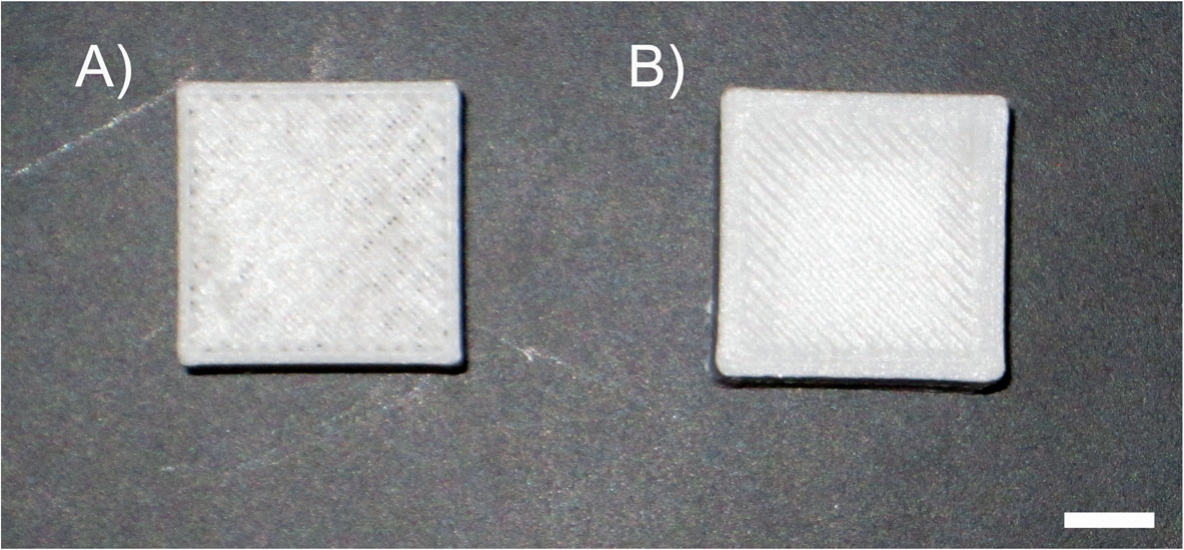
**A** A 30 x 30 x 30 mm cube printed using the Flashprint© software and the original printing method that has defects in the top layer. **B** A 30 x 30 x 30 mm cube printed using the Simplify3D® software and the other implemented changes displaying better layer deposition. Scale bar represents 10 mm

After obtaining weights and volumes for the cubes, a MARK-10® force gauge with a 1 kN load cell was used to determine the mechanical strength for the control and autoclaved cubes. Each cube was placed on the platform and a plunger with an area of 2.93 cm was lowered until it was in contact with the center of the top face of the cube. Any cubes that were not flat were discarded. The plunger was only in contact with the inner portion of the top cube face and not the thick exterior walls to better determine any change in structural integrity due to autoclaving (Figs. 1 and 3). The force was applied until structural failure or the load cell reached 995 N. The maximum of 995 N was chosen as it ensured the load cell did not go beyond its 1 kN limit. The compressive stress for each cube was found by dividing the maximum force applied by the area of the interior of the top cube face. An attached gauge was used to measure the maximum deformation experienced by the top face of each cube in millimeters. Values for the same polymer in the same group were reported as the average ± standard deviation. RStudio© was used to perform a two-way ANOVA with Tukey’s HSD on the data and *p* < 0.05 was considered statistically significant.

## Results

A Creator Pro 3D printer with FlashPrint© software was used to print rectangular objects made of PP and GFPP onto 3 in wide packing tape and several printing parameters were changed to acquire the best 3D printed rectangular objects (Table 1). Some additional problems encountered were that the brass extruders showed wear due to the abrasive nature of the GFPP filament and burning because of the high temperature needed to successfully print PP and GFPP filaments. The overlapping 3 in packing tape did not consistently adhere to the stock print bed or itself due to these same high temperatures which resulted in some non-rectangular object prints. Even when shrinkage and warpage were considerably reduced and easily identifiable rectangular objects fabricated, some small layer adhesion issues remained. It was for these reasons that the following upgrades were implemented. The extruders were changed to ones fabricated from hardened steel to better withstand the high printing temperature and abrasiveness of the GFPP filament, a larger glass printing bed was installed and covered in one layer of 7 in PP packing tape to create a more even printing surface and increase adherence, and a new printing software, Simplify3D®, was purchased because more parameters were available that increased the success of printing with PP and GFPP (Table 2). Figure 3 shows the most successful cube printed using the original printing setup with some defects in the top layer compared to a cube printed using all the implemented changes. These changes also greatly increased printing replicability and three to six cubes could be printed at once.

Table 4 displays the number of autoclaves completed, the average initial mass, the average percent mass loss, the average percent volume loss, and the melting category for PP and GFPP cubes autoclaved at 134 °C. The GFPP cubes had a slightly higher mass than the PP cubes probably due to the inclusion of glass fibers in the PP filament. PP cubes survived being autoclaved once (n = 5) and twice (n = 10), but melting was observed after the third round of autoclaving for three out of the five cubes. The average percent mass loss for PP cubes was small and slightly fluctuated because of the cubes melting. The average volume loss was less than 1 % for autoclave rounds one and two resulting in a category rating of 1, but after three rounds of autoclaving, the average volume loss increased to 1.70 % resulting in a category rating of 2. GFPP cubes also displayed a small change in mass loss with some negative values calculated that were most likely the result of the normal standard deviation affiliated with the analytical balance. GFPP cubes autoclaved at 134 °C had a larger variability as ten cubes melted after two rounds of autoclaving and two cubes out of five melted after three rounds of autoclaving. This variability was echoed in the average percent volume loss, 4.04 % ± 4.27 % after two rounds of autoclaving compared to 0.60 % ± 0.46 % after three rounds, and a category rating of 3 compared to 1 for the two and three rounds of autoclaving respectively. It was determined that this autoclave temperature was too high, so it was lowered to 121 °C and the sterilization time increased from 7 to 30 min.

**Table 4:** Values for 30 × 30 × 30 mm PP and GFPP cubes autoclaved at 134 °C. PP cubes withstood one and two rounds of autoclaving at this temperature. GFPP cubes displayed a larger variance because some cubes melted after two rounds of autoclaving, but others survived one and three rounds. Numbers in parentheses denote a negative calculated value

Polymer	Number of Autoclaves Completed	Average Initial Mass (g)	Average Percent Mass Loss	Average Percent Volume Loss	Category
PP (*n* = 5)	1	14.46 ± 0.19	0.04 % ± 0.008 %	0.33 % ± 0.34 %	1
PP (*n* = 10)	2	14.63 ± 0.20	0.01 % ± 0.007 %	0.87 % ± 0.58 %	1
PP (*n* = 5)	3	14.53 ± 0.06	0.06 % ± 0.016 %	1.70 % ± 1.81 %	2
GFPP (*n* = 5)	1	16.16 ± 0.23	(0.01)% ± 0.008 %	0.43 % ± 0.37 %	1
GFPP (*n* = 10)	2	16.06 ± 0.16	0.06 % ± 0.38 %	4.04 % ± 4.27 %	3
GFPP (*n* = 5)	3	15.91 ± 0.11	(0.03)% ± 0.03 %	0.60 % ± 0.46 %	1

The number of autoclaves completed, the average initial mass, the average percent mass loss, the average percent volume loss, and the average melting category for PP (*n* = 5/autoclave group) and GFPP (*n* = 5/autoclave group) cubes autoclaved at 121 °C are shown in Table 5. Again, the GFPP cubes had a slightly higher mass compared to the PP cubes because of the incorporated glass fibers. Both the PP and GFPP cubes displayed a very tiny change in mass after autoclaving and in some instances, a negative value was calculated due to small variations associated with the analytical balance. Autoclaved PP and GFPP cubes had less than a 1 % volumetric loss and a category rating of 1 for cubes autoclaved one, four, seven, and ten times.

**Table 5:** Values for 30 × 30 × 30 mm PP (*n* = 5/autoclave group) and GFPP cubes (*n* = 5/autoclave group) autoclaved at 121 °C. All cubes withstood up to ten rounds of autoclaving. Numbers in parentheses denote a negative calculated value

Polymer	Number of Autoclaves Completed	Average Initial Mass (g)	Average Percent Mass Loss	Average Percent Volume Loss	Category
PP	1	14.85 ± 0.03	(0.01)% ± 0.003 %	0.25 % ± 0.22 %	1
PP	4	14.61 ± 0.20	0.01 % ± 0.02 %	0.09 % ± 0.25 %	1
PP	7	14.73 ± 0.16	0.07 % ± 0.03 %	0.37 % ± 0.18 %	1
PP	10	14.67 ± 0.28	0.05 % ± 0.01 %	0.34 % ± 0.36 %	1
GFPP	1	15.81 ± 0.08	(0.03)% ± 0.01 %	0.03 % ± 0.13 %	1
GFPP	4	16.26 ± 0.22	(0.06)% ± 0.03 %	0.43 % ± 0.23 %	1
GFPP	7	16.15 ± 0.12	(0.06)% ± 0.02 %	0.10 % ± 0.56 %	1
GFPP	10	16.41 ± 0.45	(0.07)% ± 0.03 %	0.69 % ± 0.19 %	1

**Fig. 4:**
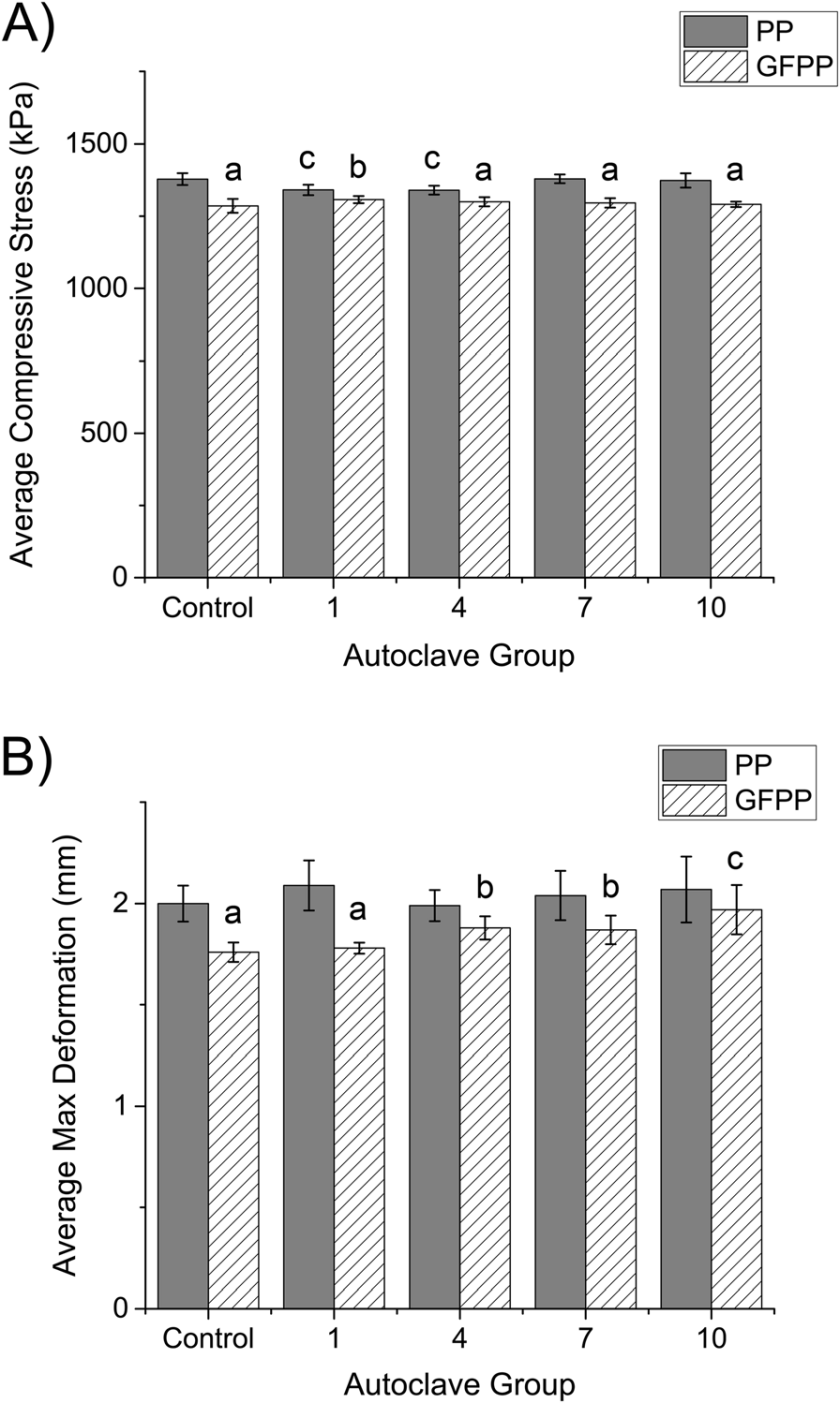
**A** Average values for compressive stress and **B** maximum deformation for the no autoclave control, one round, four rounds, seven rounds, and ten rounds of autoclaving at 121°C for 30 x 30 x 30 mm PP and GFPP cubes (*n*=5/polymer/group).^a, b, and c^indicate statistically significant differences (*p*<0.05)

All cubes withstood a max load of 995 N without structurally failing. Figure 4 shows the average compressive stress and average maximum deformation for the autoclaved PP and GFPP cubes and no autoclave controls, PP0 and GFPP0. For average compressive stress, there were no significant differences between any of the GFPP groups; however, the GFPP0, GFPP4, GFPP7, and GFPP10 groups were significantly smaller than all PP groups. The GFPP1 group was only significantly less than the PP0, PP7, and PP10 groups. In addition, the PP1 and PP4 groups were significantly less than the PP0 and PP7 groups. For average max deformation, there were no significant differences between the PP groups. The GFPP0 and GFPP1 groups deformed significantly less than all PP groups. The GFPP4 and GFPP7 groups deformed slightly more than the GFPP0 and GFPP1 groups and were only significantly less than the PP1 group. Lastly, the GFPP10 group deformed significantly more than the GFPP0 group. The GFPP cubes were stronger due to the embedded glass fibers, but the data does indicate that the printing parameters chosen for PP and GFPP fabricated mechanically strong cubes that successfully withstood up to ten rounds of autoclaving at 121 °C.

## Discussion

The goal of this project was to investigate the sustainability of 3D printed PP and GFPP rectangular objects that have undergone several rounds of steam sterilization via autoclaving to fabricate custom and/or multi-use medical devices. PP was chosen because it had the potential to survive the temperatures needed for autoclaving, but is a more difficult polymer to 3D print because it shrinks and warps during the process [13, 16, 18–20, 22, 28, 31, 32]. PP composites have been gaining traction as the rigidity and stability of PP composites reduces the shrinkage and warpage to varying degrees [13, 14, 16–19, 21, 28, 29, 31, 33, 34]. PP and GFPP were printed using a 0.4 mm brass extruder onto overlapping 3 in wide PP packing tape adhered to the stock print bed with print number 5 having the best results in comparison to prints 1–4 for fabricating 20 × 20 × 10 mm rectangular prisms (Table 1).

It is difficult to directly compare the parameters used in this study to the literature because there is a lack in consistency in the setup methodology and parameters reported. Milosevic et al., Stoof and Pickering, and Pickering and Stoof changed the print bed to a PP sheet to increase adherence to the print surface and printed single extruded rows of PP composites at 230 °C with a 1, 1.5, or 2 mm die at a rate of 50 mm/min [13, 14, 17]. Neat PP was shown to shrink and warp by Pickering and Stoof, but the addition of hemp or Harakeke fibers reduced the shrinkage and warpage, a larger diameter die was used to prevent clogging, a slower printing rate, and a lower print head temperature were used [13, 14, 17]. Long et al. combined PP, PLA, and bamboo fibers to increase the mechanical properties compared to PLA alone. This composite was 3D printed at speeds between 40 and 60 mm/s, had a print head temperature between 180 and 200 °C, and a print platform temperature between 40 and 60 °C [33]. While the PP and GFPP print speed falls in this range, the print head temperature and platform temperature were lower which was attributed to the addition of PLA into the composite. Kabiri et al. 3D printed PP containing long glass filled yarns (PPLGFY) through a 0.4 mm extruder at 220 °C at a printing speed of 5 mm/s and a layer height of 0.22 mm. Though the same extruder diameter and layer height were used, the printing speed was much slower, the printing head temperature was lower, and it was determined that a fixation plate made from PP containing long glass fibers (PPLGF) created using a heat compressing process was better than the 3D printed PPLGFY one [29].

In order to continue to increase the successfulness of 3D printing with PP and GFPP filament, several additional changes were made. These included using a 0.6 mm hardened steel extruder, installing a larger glass print bed, changing from 3 in PP packing tape to 7 in PP packing tape because one single layer would cover the printing surface and provide better adhesion, employing a raft to decrease shrinkage and warpage, using Simplify3D® software, and printing in an enclosed chamber. Figure 3 displays the difference in the best 30 × 30 × 30 mm cube printed using the parameters for print 5 (Table 1) compared to a cube printed using these new changes (Table 2).

Several manuscripts also used Simplify3D® software to 3D print PP and PP composites [18, 19, 21, 22, 34]. Several manuscripts by Spoerk et al. also used a 0.6 mm steel nozzle, but the print head temperature was 220 or 230 °C, the print bed temperature was 20 or 70 °C, the layer height was 0.25 mm, and it was printed on a PP plate bed [18, 19, 21]. In comparison to the second method used in this study, the print head and bed temperatures were lower, but the layer height was greater. However, the addition of the composite filler helped reduce the shrinkage and warpage seen when printing PP only [18, 19]. Bachhar et al. printed isotactic PP (iPP) through a 0.6 mm extruder at 230 °C onto a glass surface covered with a polyvinyl acetate-based adhesive at 60 °C with a print speed of 30 mm/s, the top and bottom thickness were 1 mm, square lattice infills of 20 %, 50 %, or 100 % were used, and no brim, a 4.5 mm brim, or a 13.5 mm brim were printed prior to fabricating the 50 × 15 × 10 mm rectangular bars. The adhesive allowed the iPP to adhere well to the glass print surface and the brim was a single printed layer used to stabilize the object during printing, but was removed before testing. The use of a brim and the 20 % infill amount reduced the iPP warpage which was similar to the 25 % infill and raft printed using PP and GFPP [22]. These studies still observed some degree of warpage and shrinkage when printing using PP and/or PP composites; however, the second methodology used in this study effectively alleviated shrinkage and warpage for both PP and GFPP printed cubes.

Reusability of custom 3D printed medical devices could diminish the amount of medical waste by being able to survive several rounds of steam sterilization by autoclaving. PP and GFPP cubes were autoclaved at 134 and 121 °C one, four, seven, and ten times and compared to a no autoclave control. PP and GFPP cubes did not consistently survive being autoclaved at 134 °C after two or three rounds; however, lowering the temperature to 121 °C enabled all cubes to be autoclaved up to ten times with no melting observed and minimal percent volume loss (Tables 4 and 5).

For mechanical testing, the majority of the literature focuses on tensile testing rather than compressive testing because single fibers or objects with much smaller heights were 3D printed to decrease the shrinkage and warpage that occurs as more layers are 3D printed [13, 14, 16, 17, 28, 33, 34]. Although the average compressive stress values were very similar for the PP and GFPP cubes, the GFPP0, GFPP4, GFPP7, and GFPP10 cubes were significantly smaller compared to all PP ones. The only exception was the GFPP1 group since, it was only significantly less than the PP0, PP7, and PP10 groups. There were no significant differences between the GFPP groups and the only difference in the PP groups was that the PP1 and PP4 groups were significantly less than the PP0 and PP7 groups (Fig. 4 A). For the average max deformation, the GFPP0 and GFPP1 groups deformed less compared to all PP groups, but the GFPP4 and GFPP7 groups were only significantly smaller compared to the PP1 group (Fig. 4B). While the embedded glass fibers provided additional strength, the GFPP0 group had significantly less deformation than the GFPP10 group. In addition, there were no significant differences between the PP groups. These discrepancies could be explained by print-to-print variation or inconsistent amounts of glass fibers embedded in the PP filament. Kabiri et al. found that the ultimate compressive strength for PP was 20 ± 5 MPa compared to 30 ± 5 MPa, 15 ± 5 MPa, and 15 ± 5 MPa for PPLGFY in each of the three directions tested [29]. Ultimate compressive stress was unable to be determined in this study as the load cell was maxed out prior to material failure.

## Conclusions

The overall goal was to successfully 3D print rectangular objects using PP and GFPP filaments and test their ability to survive up to ten rounds of autoclaving without melting or displaying a decrease in mechanical strength. The best printing setup included an enclosed glass print bed with 7 in PP packing tape, a 0.6 mm hardened steel extruder, printing a sacrificial raft first, and the use of Simplify3D® software because it allowed for more specific printing parameters to be controlled. This procedure resulted in the ability to print between three to six cubes at one time with consistent replicability. An initial autoclave temperature of 134 °C was found to be too high because it caused several of the PP and GFPP cubes to melt after two to three rounds of autoclaving, so the temperature was lowered to 121 °C for the remainder of the study. The hypothesis for this study was rejected as a single procedure was found to successfully 3D print rectangular objects using both PP and GFPP and neither material melted after being autoclaved up to ten times. There were also differences in the average compressive stress and average max deformation between the two polymer groups. The GFPP0, GFPP4, GFPP7, and GFPP10 groups were significantly less than all PP groups for compressive stress; however, the GFPP1 group was only significantly less than the PP0, PP7, and PP10 groups. Also, the GFPP0, GFPP1, GFPP4, and GFPP7 groups deformed less compared to the PP groups. These results are most likely due to the embedded glass fibers providing additional strength. Future studies should focus on the mechanical properties of the PP and GFPP printed cubes using a larger load cell, increasing the sample size, and increasing the number of autoclave rounds past ten to continue developing methods to create custom and/or more multi-use medical devices to reduce both cost and waste.

## Data Availability

The datasets used and/or analyzed during the current study are available from the corresponding author on reasonable request.

## Abbreviations

PP: Polypropylene
GFPP: Glass filled PP
WHO: World Health Organization
FDM: Fused deposition modelling
FFF: Fused filament fabrication
PLA: Poly(lactic acid)
ABS: Acrylonitrile butadiene styrene
*m*_*i*_: Initial mass
*v*_*i*_: Initial volume
*m*_*f*_: Final mass
*v*_*f*_: Final volume
Eq.: Equation
PPLGFY: Polypropylene with long glass fibers
iPP: Isotactic polypropylene
VAS: Virginia Academy of Sciences
